# Quantifying Human Movement Using the Movn Smartphone App: Validation and Field Study

**DOI:** 10.2196/mhealth.7167

**Published:** 2017-08-17

**Authors:** Ralph Maddison, Luke Gemming, Javier Monedero, Linda Bolger, Sarahjane Belton, Johann Issartel, Samantha Marsh, Artur Direito, Madeleine Solenhill, Jinfeng Zhao, Daniel John Exeter, Harshvardhan Vathsangam, Jonathan Charles Rawstorn

**Affiliations:** ^1^ Institute for Physical Activity and Nutrition School of Exercise and Nutrition Sciences Deakin University Burwood Australia; ^2^ National Institute for Health Innovation School of Population Health University of Auckland Auckland New Zealand; ^3^ School of Health & Human Performance Dublin City University Dublin Ireland; ^4^ Center of Research on Welfare Health and Sport School of Health and Welfare Halmstad University Halmstad Sweden; ^5^ Department of Epidemiology and Biostatistics School of Population Health University of Auckland Auckland New Zealand; ^6^ Robotic Embedded Systems Laboratory Robotics and Autonomous Systems Center University of Southern California Los Angeles, CA United States

**Keywords:** telemedicine, smartphone, validation studies, geographic information systems, locomotion, physical activity, humans

## Abstract

**Background:**

The use of embedded smartphone sensors offers opportunities to measure physical activity (PA) and human movement. Big data—which includes billions of digital traces—offers scientists a new lens to examine PA in fine-grained detail and allows us to track people’s geocoded movement patterns to determine their interaction with the environment.

**Objective:**

The objective of this study was to examine the validity of the Movn smartphone app (Moving Analytics) for collecting PA and human movement data.

**Methods:**

The criterion and convergent validity of the Movn smartphone app for estimating energy expenditure (EE) were assessed in both laboratory and free-living settings, compared with indirect calorimetry (criterion reference) and a stand-alone accelerometer that is commonly used in PA research (GT1m, ActiGraph Corp, convergent reference). A supporting cross-validation study assessed the consistency of activity data when collected across different smartphone devices. Global positioning system (GPS) and accelerometer data were integrated with geographical information software to demonstrate the feasibility of geospatial analysis of human movement.

**Results:**

A total of 21 participants contributed to linear regression analysis to estimate EE from Movn activity counts (standard error of estimation [SEE]=1.94 kcal/min). The equation was cross-validated in an independent sample (N=42, SEE=1.10 kcal/min). During laboratory-based treadmill exercise, EE from Movn was comparable to calorimetry (bias=0.36 [−0.07 to 0.78] kcal/min, *t*_82_=1.66, *P*=.10) but overestimated as compared with the ActiGraph accelerometer (bias=0.93 [0.58-1.29] kcal/min, *t*_89_=5.27, *P*<.001). The absolute magnitude of criterion biases increased as a function of locomotive speed (*F*_1,4_=7.54, *P*<.001) but was relatively consistent for the convergent comparison (*F*_1,4_=1.26, *P*<.29). Furthermore, 95% limits of agreement were consistent for criterion and convergent biases, and EE from Movn was strongly correlated with both reference measures (criterion *r*=.91, convergent *r*=.92, both *P*<.001). Movn overestimated EE during free-living activities (bias=1.00 [0.98-1.02] kcal/min, *t*_6123_=101.49, *P*<.001), and biases were larger during high-intensity activities (*F*_3,6120_=1550.51, *P*<.001). In addition, 95% limits of agreement for convergent biases were heterogeneous across free-living activity intensity levels, but Movn and ActiGraph measures were strongly correlated (*r*=.87, *P*<.001). Integration of GPS and accelerometer data within a geographic information system (GIS) enabled creation of individual temporospatial maps.

**Conclusions:**

The Movn smartphone app can provide valid passive measurement of EE and can enrich these data with contextualizing temporospatial information. Although enhanced understanding of geographic and temporal variation in human movement patterns could inform intervention development, it also presents challenges for data processing and analytics.

## Introduction

The World Health Organization (WHO) recognizes physical inactivity as one of the leading global risk factors for morbidity and premature mortality [1]. Despite the considerable benefits of regular physical activity (PA; [2,3]), it has been estimated that 21.4% of the global population is inactive (perform little or no activity), with greater prevalence of physical inactivity among most developed countries (27.8%; [4]).

To move forward in PA research, it has been suggested that “more of the same is not enough” [5]. Different approaches are needed to reduce the burden of disease associated with physical inactivity. Technological innovations such as smartphones and wearable sensors offer potential to improve the reach, enhance delivery (greater frequency of contact and duration of intervention), and increase effectiveness of interventions to improve PA levels. Despite their potential, it is unclear whether these new devices provide research-grade precision measurement. To address this concern, a number of validation studies have been conducted [6-8].

Compared with the frequently used ActiGraph accelerometer, studies have demonstrated acceptable levels of agreement over periods of 7 days against the Fitbit Zip wearable sensor [6] and CalFit smartphone app [8]. Despite the acceptable measurement properties of new wearable sensors, the benefit of smartphones is that they are carried by most people, most of the time. At the population level, their ubiquitous use, the big data (millions of data points) and geospatial information generated by smartphones may offset any potential measurement inaccuracies by providing valuable insight into behavioral patterns (eg, temporal stability) and their contexts. Moreover, the potential of these technologies are vast. For example, coupled with the Internet of Things [9], smartphones can be used to track people’s movement within cities or environments, thereby providing a rich source of contextual information and the potential to deliver “just-in-time” interventions. Compared with stand-alone accelerometers, smartphones offer advantages in terms of usability and integration of supplementary data. These features support the delivery of more responsive, engaging, and context-specific interventions that could improve uptake, adherence, and effectiveness.

Few published studies have explored the utility of smartphones to measure both PA and human movement. A recent convergent validity study comparing an Android smartphone activity tracker against the ActiGraph accelerometer found acceptable associations and agreement in both laboratory and free-living environments [10]. To extend the evidence, further validation work against criterion measures is required [11]. Furthermore, health is geospatial, and if we can see trends in behavior spatially, we can monitor (and improve) population and individuals’ needs [12]. To illustrate, González et al [13] used anonymized cellular phone data from 100,000 users in New York, United States, to capture people’s position over a period of 6 months. They showed that human trajectories had a high degree of temporal and spatial regularity. In other words, humans follow simple reproducible patterns; this in turn has important implications for interventions to enhance human mobility [13]. In PA research, the feasibility of linking global positioning system (GPS) and accelerometer data has been well established [14-16]. However, these studies have typically involved the use of 2 separate devices (accelerometer and GPS) for limited periods (7-28 days). Smartphone apps offer advantages over these approaches; they are relatively cheap, readily available, incorporate native sensors (GPS, gyroscopes, and accelerometers), and permit passive data collection—thus requiring minimal input from participants. This has clear advantages in terms of reducing participant burden for research.

We aimed to examine the validity of the Movn smartphone app for estimating PA energy expenditure (EE) and quantifying human movement patterns. Two validation studies were conducted against criterion and convergent methods; a supporting cross-validated study assessed the consistency of activity data when collected across different devices. GPS and geographic information system (GIS) data were integrated to demonstrate the feasibility of geospatial analysis.

## Methods

A dual-phase cross-sectional study was conducted to determine the validity of the Movn smartphone app (Moving Analytics) for assessing EE and human movement patterns during laboratory-based and free-living daily activities among a convenience sample of healthy adults. Phase 1 comprised laboratory-based treadmill exercise at light to vigorous levels of intensity and free-living daily activities. Phase 2 comprised a cross-validation during laboratory-based activities among a separate sample of participants. EE was the main measurement of interest; it is the most appropriate outcome for validating accelerometers as it can be directly related to the accepted methods of PA categorization, compared with robust criterion data collected via indirect calorimetry, and allows standardized comparison with other accelerometer devices [17].

### Study Participants and Recruitment

In phase 1, a total of 21 adults (13 female), aged 20 to 55 years, were recruited in Dublin, Ireland (see Table 1). Participants were recruited via direct contact through the university and by word of mouth. Adults were eligible for inclusion provided they met the following criteria: aged 18 to 65 years, able to give written informed consent, and able to communicate in English. In Phase 2, a total of 42 adults (27 male) aged between 18 and 33 years were recruited from the Greater Los Angeles Area. Participants were recruited via direct contact through the University of Southern California and by word of mouth. Phase 1 and 2 study protocols were approved by the Dublin City University Research Ethics Committee and the institutional review board of the University of Southern California, respectively.

### Phase 1 Procedures

Upon arrival to the laboratory, participants completed demographic information, including age and sex, and the Physical Activity Readiness Questionnaire [18]; all were deemed safe to exercise. Anthropometric measurements were taken; height was measured to the nearest 0.1 cm with a portable stadiometer, and weight was measured to the nearest 0.1 kg on an electronic scale (Seca).

Reference accelerometry was quantified using the GT1M (ActiGraph Corp), a dual-axis accelerometer with established reliability and validity [19]. Epoch duration was set to 1 s during laboratory-based activities and 10 s during free-living activities. ActiGraph devices were fitted to an elastic belt on participants’ right hip at the midaxillary line for the duration of the test. Comparison accelerometry was quantified using the Moto G first-generation smartphone (Motorola Mobility LLC) running Android version 4.3 (Google Inc) and a research version of the Movn app. Movn is a commercially available app—for Android and Apple iPhone operating systems—that uses inbuilt smartphone accelerometers to passively quantify time spent in moderate-to vigorous-intensity physical activities such as walking and running. Raw accelerometer data were captured at the maximum frequency permitted by the phone hardware (at/above 200 Hz) and downsampled to 50 Hz. The Movn app samples GPS data every 30 min unless movement is detected, and every minute during periods of movement; this sampling approach was adopted to balance sampling frequency and power consumption. Movn also allows users to set daily PA goals and can issue prompts throughout the day to facilitate goal achievement. The smartphone was secured in a phone holder and positioned adjacent to the ActiGraph accelerometer. The same phone was used for all the participants.

Following familiarization with the smartphone and accelerometer, the K4b2 portable indirect calorimeter (Cosmed) was used to assess resting and exercise EE [20]. A 2-point calibration procedure was conducted before each testing session according to the manufacturer’s guidelines. Calibration of the oxygen (O_2_) and carbon dioxide (CO_2_) sensors was performed with standard gases of known concentrations (gas 1: O_2_=20.93%, CO_2_=0.04%; gas 2: O_2_=15.00%, CO_2_=5.00%). Respiratory volume was calibrated using a 3-L syringe. The rate of EE was estimated using the following formula as calculated by the K4b2 system: EE (kcal/min)=(3.781 × V̇O_2_) + (1.237 × V̇CO_2_) if UN (urea nitrogen)=0, where V̇O_2_=oxygen uptake (L/min) and V̇CO_2_=carbon dioxide production (L/min) [20].

A face mask (Hans Rudolf) held in place by a nylon harness covered the participants’ nose and mouth. The mask was attached to a bidirectional digital turbine flow meter to measure the volumes of inspired and expired air. Heart rate data were captured using the FT1, a chest-worn sensor (Polar Electro Oy).

Once instrumented, participants remained seated for 15 min while physiological data were recorded; the last 5 min of data were averaged to calculate resting EE. Participants then completed four discrete bouts of walking and running on a motorized treadmill (Quasar Med, H/P Cosmos Sports & Medical GmbH). Participants completed 5-min bouts of exercise at light (4 km/h and 6 km/h), moderate (10 km/h), and vigorous intensity levels (≥12 km/h), separated by 3-min bouts of passive recovery.

Following laboratory-based activities, participants were instructed to wear the ActiGraph accelerometer (as described above) and carry the smartphone (as they would normally carry their personal phone) for 24 hours during free-living daily activities. Participants were instructed to remove the devices during water-based activities such as swimming, showering, and bathing. An optional sports armband carry case was provided to participants who preferred this carry method during free-living exercise. After 24 hours, participants returned the phone and accelerometer.

### Phase 2 Procedures

Procedures for the cross-validation sample have been described elsewhere [21]. In brief, each participant wore a Samsung Galaxy Nexus S phone, Android version 2.3.3 with Movn app, installed on the right iliac crest with a belt holder to record movement. EE was measured by the Oxycon portable indirect calorimeter (CareFusion) worn in a backpack fitted to the comfort of the participant. Participants completed three 6-min bouts of treadmill walking (4, 5, and 6 km/h), separated by 2 min of passive recovery.

### Data Handling

Heart rate, calorimeter, and ActiGraph data were downloaded using the manufacturers’ software; smartphone data were downloaded using a text reader, exported for manual analysis, and synchronized in postprocessing.

Mean values were calculated during the last 2 min of each laboratory-based activity bout, following similar procedures in phase 1 and 2. Free-living accelerometer data were cleaned by removing nonwear time, equivalent to >60 min of continuous zero counts. A minimum of 10 hours of available free-living data were required for the analysis. Data were processed to generate comparable units of EE for analysis [17].

### Statistical Analysis

Statistical analyses were performed using the Statistical Package for the Social Sciences (SPSS) version 21 for Windows (IBM Corp).

To derive estimates of EE from Movn activity counts, multivariate regression was conducted following established methods [19] to identify the strongest relationship between Movn activity counts, participant characteristics, and EE measured via indirect calorimetry. Estimates of EE were then used to identify Movn activity count thresholds associated with accepted classifications for light (<3 metabolic equivalent of task [MET]), moderate (3-6 MET), hard (6-9 MET), and very hard (>9 MET) activity intensity levels [19]. The regression equation was also applied to phase 2 laboratory-based data to cross-validate the accuracy of EE estimation among an independent sample using different smartphone hardware.

EE measurement validity was evaluated following guidelines proposed by Welk et al [17], who suggest that agreement between two measurement methods requires demonstration of three unique characteristics: equivalent group estimates, association between measurements, and absence of systematic and/or heterogeneous bias. Furthermore, supplementary analyses at an individual level are also recommended to determine whether group-level agreement is consistent across individuals.

The criterion and convergent validity of Movn EE measurement were assessed compared with EE measured via indirect calorimetry during phase 1 laboratory-based activities and with EE estimated from ActiGraph movement counts during phase 1 laboratory and free-living activities, respectively; *t*-tests were conducted to detect systematic criterion and convergent group-level measurement biases. Simple analyses of variance were also conducted to determine whether group-level criterion or convergent measurement biases were affected by activity intensity level during laboratory-based activities; significant main effects were explored with Bonferroni-corrected paired comparisons (ie, least significant difference *P* × N(N−1)/2 paired comparisons). Furthermore, 95% limits of agreement for biases were calculated to assess absolute measurement agreement and homogeneity of biases across the measurement range [22,23]. Relationships between Movn and reference measurement methods were assessed by calculating Pearson correlation coefficients and two-way random effects intraclass correlation coefficients for absolute agreement (ICC).

Supplementary analyses compared time-synchronized group-level measurements throughout the laboratory-based activity protocol to determine agreement between measurement patterns [17]. Furthermore, individual-level biases were calculated to determine whether group-level agreement was consistent across the sample.

To determine the feasibility of using smartphone sensor data to ascertain the geographic location of activity and patterns of human movement, accelerometer and GPS data were combined to provide an indication of the location and intensity of PA. Data were imported into ArcGIS version 10.2.2 (Esri) transformed to location points and interpolated into two-dimensional (2D) spatial paths and three-dimensional (3D) spatiotemporal trajectories.

Descriptive data are reported as mean (standard deviation); bias data are reported as mean and 95% CI; alpha=.05 for all hypothesis tests.

## Results

Phase 1 participants included 21 adults (13 female) aged 20 to 55 years (see Table 1); phase 2 participants included 42 adults (27 male) aged 18 to 33 years. All participants were at normal weight.

### Estimating Energy Expenditure

**Figure 1:**
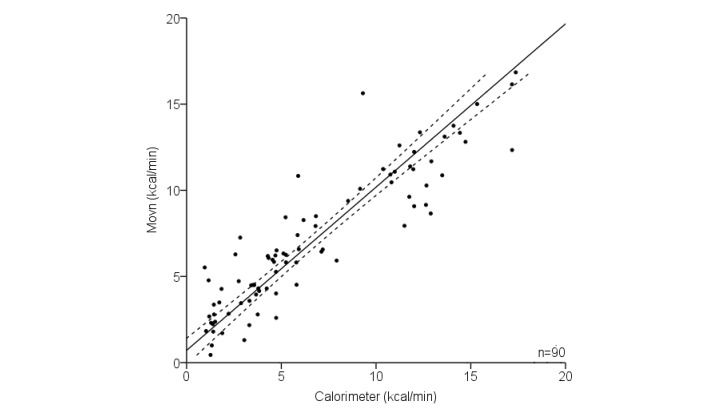
Relationship between measured energy expenditure (EE; indirect calorimetry) and Movn app EE derived from multivariate regression of Movn activity counts on measured EE during phase 1 laboratory-based activities.

A multivariate regression model including Movn activity counts and participant body mass was the strongest predictor of measured EE (Equation 1, *r*^2^=.83; SEE=1.94 kcal/min). The relationship between measured and predicted EE is shown in Figure 1.

Equation 1: EE (kcal)=0.00063 × activity level (counts/min) + 0.121 × body mass (kg) − 5.66

Equation 1 was used to identify Movn activity count thresholds that correspond with accepted classifications of light (<3 MET), moderate (3-6 MET), hard (6-9 MET), and very hard (≥9 MET) activity intensity levels [19]; thresholds derived from phase 1 laboratory-based data are presented in Table 2. A correction factor (2121) was applied to the smartphone accelerometer data to facilitate scale congruence and allow comparison between Movn and ActiGraph activity counts.

When applied to the cross-validation sample (phase 2), Equation 1 estimation accuracy was lower during laboratory-based walking (*r*^2^=.24; SEE=1.10 kcal/min).

**Table 1:** Participant characteristics.

Demographics	Phase 1 Mean (SD)	Phase 2 Mean (SD)
N (men/women)	21 (8/13)	42 (27/15)
Age, in years	27 (7.9)	26 (3.8)
Height (cm)	171.2 (7.3)	172 (8)
Weight (kg)	70.5 (11.6)	68 (12.0)
Body mass index (kg/m^2^)	23.1 (2.6)	22.0 (3.0)

**Table 2:** Movn activity count thresholds for classifying activity intensity level.

Activity intensity level^a^	MET^b^range	Activity (counts/min)
Light	<3	<1253
Moderate	3-6	1253-1272
Hard	6-9	1273-6987
Very hard	≥9	>6987

^a^Activity intensity level classification adapted from Freedson et al [19].

^b^MET: metabolic equivalent of task.

### Laboratory-Based Activities

#### Criterion Validity

Table 3 summarizes EE during phase 1 laboratory-based treadmill exercise, assessed with indirect calorimetry, Movn, and ActiGraph. Movn overestimated EE compared with the criterion indirect calorimetry method (Table 3), but the small magnitude did not represent a systematic measurement bias (*t*_82_=1.66, *P*=.10). A statistically significant main effect of activity intensity level was detected on measurement biases (*F*_1,4_=7.54, *P*<.001), indicating systematic variance across laboratory-based activity levels. The Movn app overestimated EE at rest and slower locomotive speeds and underestimated EE at faster locomotive speeds (Table 3). Bonferroni-corrected paired comparisons revealed that the absolute EE measurement bias at 12 km/h was statistically significantly larger than all other speeds with the exception of 10 km/h. Furthermore, the EE measurement bias at 10 km/h was statistically significantly smaller than during rest.

**Figure 2:**
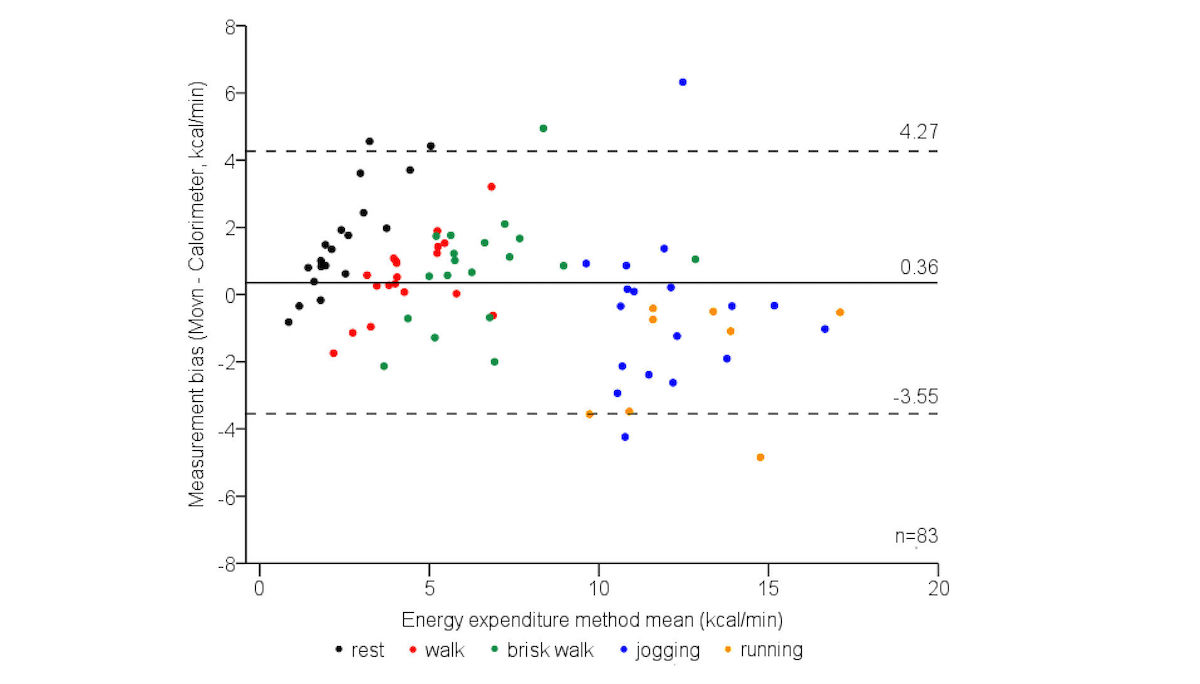
The 95% limits of agreement for phase 1 laboratory-based criterion energy expenditure measurement biases, categorized by activity intensity level.

The 95% limits of agreement for criterion EE measurement biases were moderate at most activity intensity levels (Figure 2), indicating acceptable absolute measurement agreement. Biases were relatively consistent across the measurement range; however, variance was slightly wider at faster locomotive speeds (10-12 km/h, Table 3). Finally, Movn and criterion EE measures were strongly correlated (*r*=.91, ICC=.95, both *P*<.001), indicating excellent relative measurement agreement.

#### Convergent Validity

Compared with EE derived from the convergent reference ActiGraph device during phase 1 laboratory-based activities, Movn systematically overestimated EE (*t*_89_=5.27, *P*<.001, Table 3). There was no statistically significant main effect of activity intensity level (*F*_1,4_=1.26, *P*<.29), indicating convergent measurement biases were relatively consistent across laboratory-based activity levels.

**Figure 3:**
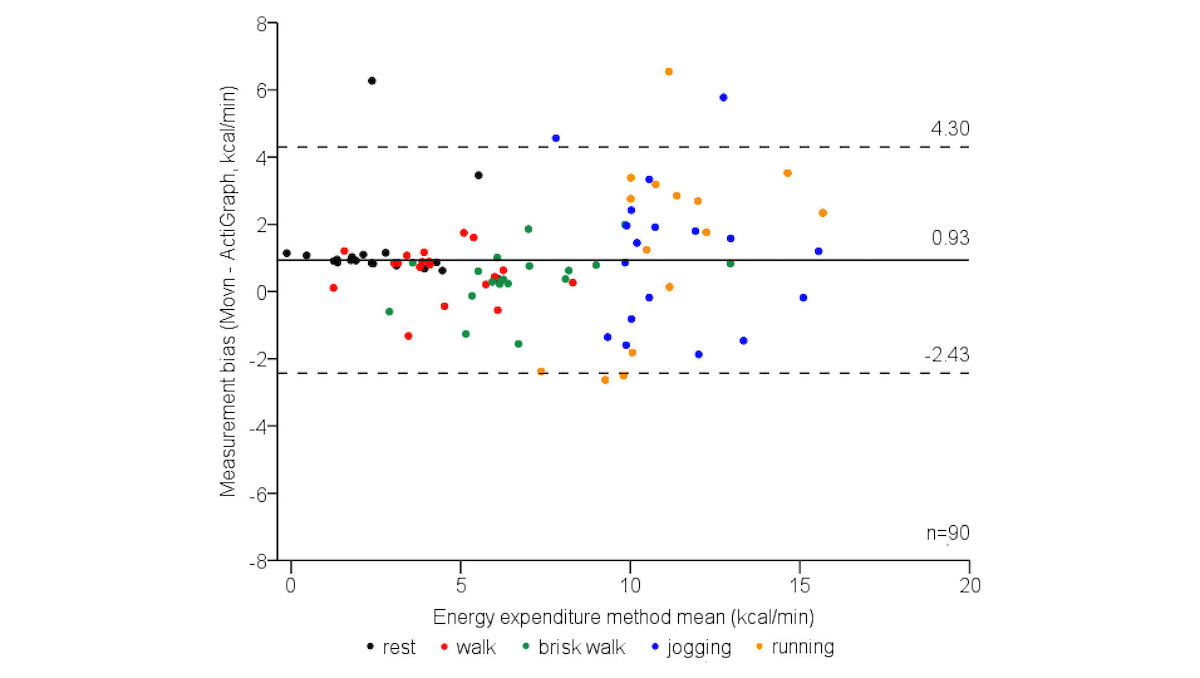
The 95% limits of agreement for phase 1 laboratory-based convergent energy expenditure measurement biases, categorized by activity intensity level.

The 95% limits of agreement for convergent EE measurement biases were also moderate at all activity intensity levels (Figure 3), indicating acceptable absolute measurement agreement. Once again, measurement error was relatively consistent across the measurement range; however, similar to the criterion analysis, variance was slightly wider at faster locomotive speeds (10-12 km/h, Table 3). Finally, Movn and criterion EE measures were strongly correlated (*r*=.92, ICC=.93, both *P*<.001), indicating excellent relative measurement agreement.

**Table:** 

Activity	Energy expenditure (kcal/min)				Biases (kcal/min)	
	Calorimeter	Movn	ActiGraph	Criterion		Convergent
	Mean (SD)				Mean (95% CI)	
Rest	1.75 (0.64)	3.25 (1.80)	1.83 (1.68)	1.60 (0.85-2.35)^d,e^		1.30 (0.65-1.96)
4 km/h	4.15 (1.20)	4.66 (1.69)	4.10 (1.83)	0.52 (−0.04 to 1.08)^e^		0.59 (0.22-0.95)
6 km/h	6.42 (2.13)	6.95 (2.45)	6.67 (2.30)	0.74 (−0.05 to 1.52)^e^		0.40 (−0.02 to 0.82)
10 km/h	11.94 (2.22)	11.78 (2.18)	10.26 (2.52)	−0.53 (−1.67 to 0.60)^a^		1.08 (0.01-2.15)
12 km/h	14.15 (2.68)	11.76 (2.97)	10.87 (1.86)	−1.90 (−3.37 to −0.42)^a-c^		1.05 (−0.81 to 2.92)
Total	7.08 (4.79)	7.45 (4.15)	6.54 (3.90)	0.36 (−0.07 to 0.78)		0.93 (0.58-1.29)^f^

^a-e^Systematic difference in bias between locomotive speeds (*P*<.001-.01, Bonferroni-corrected).

^a^Rest.

^b^4km/h.

^c^6km/h.

^d^10km/h.

^e^12km/h.

^f^Overall systematic bias compared with the ActiGraph device (*P*<.001).

Energy expenditure=average during third and fourth min of each intensity bout.

### Supplementary Analyses

**Figure 4:**
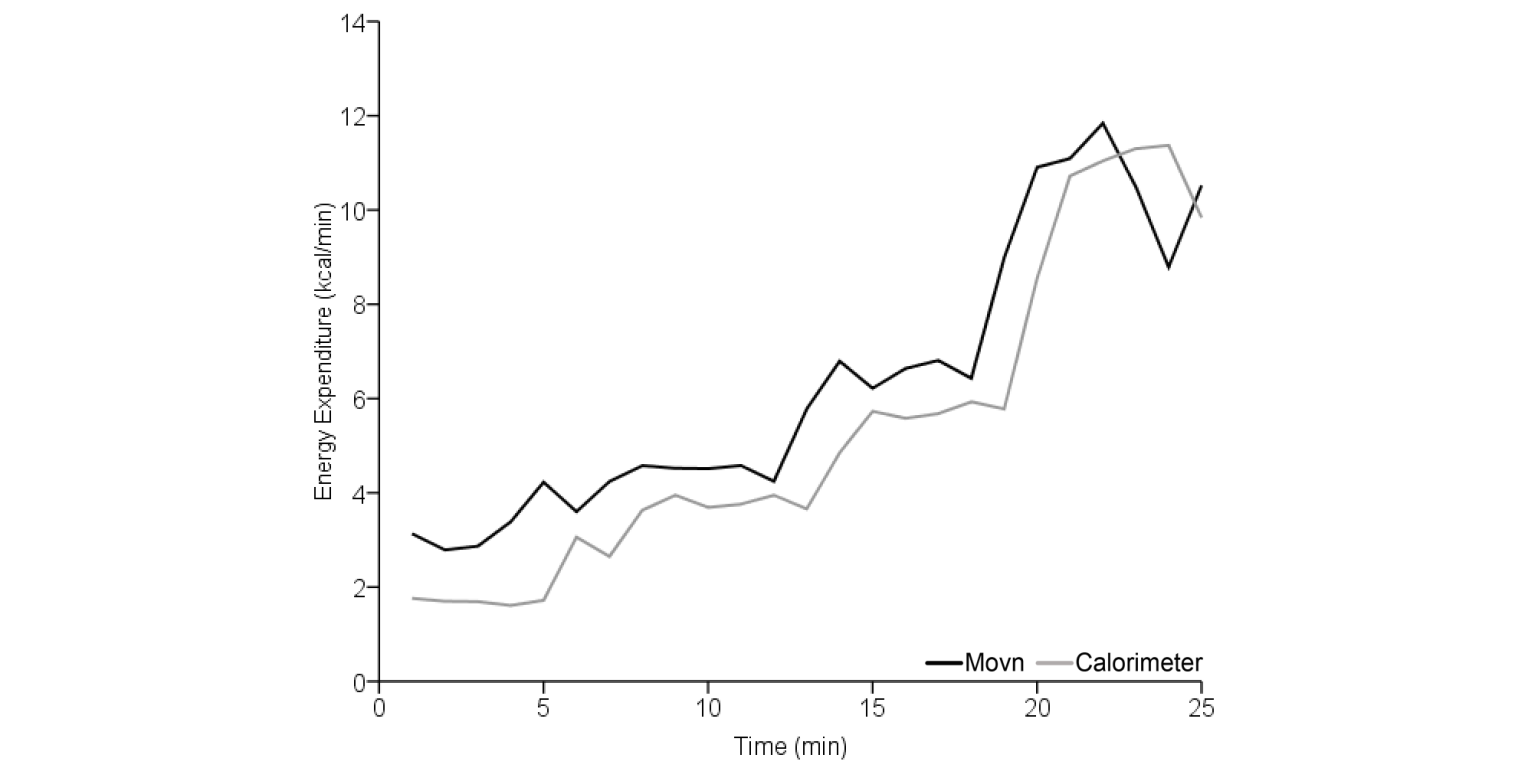
Supplementary analysis of the relative accuracy of measured (indirect calorimetry) and estimated (Movn app) energy expenditure (EE) during phase 1 laboratory-based activities. Minute-by-minute EE measured by the Movn app and criterion reference calorimeter.

Supplementary analyses comparing time-synchronized group-level measurements throughout the phase 1 laboratory-based activity protocol indicate that Movn and calorimetry EE measurement patterns were very similar at low and moderate activity intensity levels (Figure 4; [17]); however, two important trends were identified. First, the Movn EE measurement pattern diverts substantially below the criterion calorimetry measurement late in the exercise protocol during the fastest locomotive speeds; differing measurement patterns at faster speeds indicate that the Movn app may be less valid for quantifying high-intensity locomotive activities. Second, notable asynchronicity between Movn and calorimeter EE measurement patterns reflects the expected latency between changes in energy demands (ie, instant change in locomotive speed) and physiological EE (ie, gradual increase in oxygen consumption).

**Figure 5:**
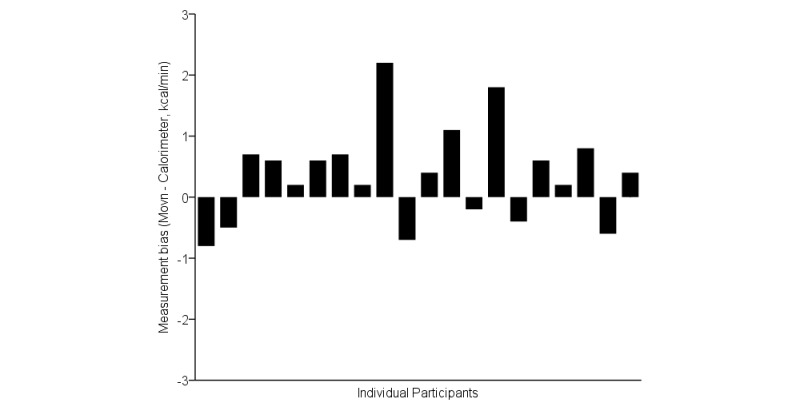
Supplementary analysis of the relative accuracy of measured (indirect calorimetry) and estimated (Movn app) energy expenditure (EE) during phase 1 laboratory-based activities. Mean EE measurement biases across individual participants.

Examination of individual-level measurement biases revealed relatively small overestimation of total EE among the majority of participants; however, EE was substantially overestimated for 2 participants and underestimated for 6 participants (Figure 5; [17]). This inconsistency suggests Movn may have more utility for group-level surveillance tool than individual-level measurement.

### Free-Living Activities

#### Convergent Validity

**Figure 6:**
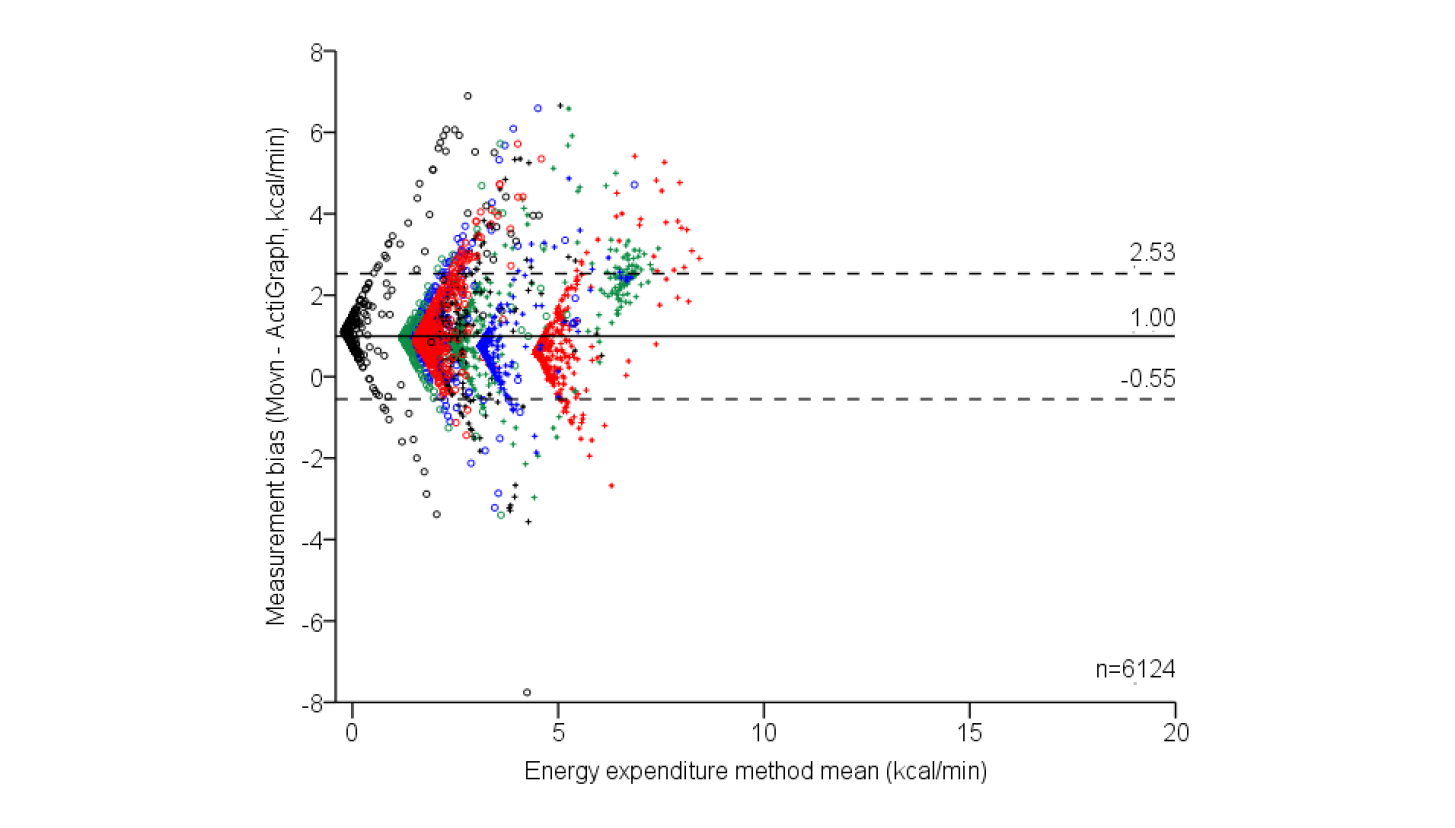
The 95% limits of agreement for phase 1 free-living convergent energy expenditure measurement biases; categorized by individual participants who recorded ≥10 hours activity data per day.

Sensor wear time compliance was lower than anticipated during phase 1 free-living activities. After removing nonwear time, only 8 participants met minimum data requirements. Participants recorded an average of 766 (SD 189) min of activity on valid days, yielding a total free-living convergent reference sample of 6124 min. Table 4 summarizes EE during phase 1 free-living activities, assessed with Movn and ActiGraph devices. Compared with the convergent reference ActiGraph, Movn overestimated EE during free-living activities (Table 4); the mean bias was larger than during laboratory-based activity and represented a systematic bias between devices (*t*_6123_=101.49, *P*<.001). Consistent with laboratory-based activities, a statistically significant effect of activity intensity level was also detected on free-living measurement biases (*F*_3,6120_=1550.51, *P*<.001), indicating systematic variance in measurement biases across commonly used levels for classifying free-living activity intensity (Table 2). Movn overestimated EE during all free-living activity levels and, with the exception of light- and moderate-intensity activity levels, bias magnitudes grew with activity intensity level (Table 4). Heterogeneous measurement biases during free-living activity are presented in Figure 6, which has been graphically categorized by individual participant (n=8) to highlight both the variance in resting EE, and relatively consistent pattern of bias heterogeneity between individuals. Positive and negative biases appear relatively symmetrical within participants (Figure 6). Further investigation indicates negative measurement biases may reflect periods of smartphone noncarry time; however, as it was not possible to validate this assumption, we conservatively treated the data as measurement error.

Finally, despite the systematic bias, EE measures from Movn and ActiGraph were strongly correlated (*r*=.87, ICC=.83, both *P*<.001), indicating excellent relative measurement agreement during free-living activities.

**Table 4:** Energy expenditure during phase 1 free-living activities.

Level of intensity	Energy expenditure (kcal/min)		Bias (kcal/min)
	Movn Mean (SD)	ActiGraph Mean (SD)	Mean (95% CI)
Light (<3 metabolic equivalent of task [MET])	2.43 (1.15)	1.61 (1.34)	0.83 (0.81-0.84)^c,d^
Moderate (3-6 MET)	2.32 (0.86)	1.01 (0.65)	1.31 (0.69-1.93)^c,d^
Hard (6-9 MET)	4.19 (1.49)	2.24 (1.6)	1.96 (1.87-2.04)^a,b,d^
Very hard (≥9 MET)	7.66 (1.19)	4.25 (2.01)	3.41 (3.16-3.66)^a-c^
Total	2.73 (1.51)	1.73 (1.45)	1.00 (0.98-1.02)^e^

^a-d^Systematic difference in biases between activity intensity levels (*P*<.001-.02, Bonferroni-corrected).

^a^Light.

^b^Moderate.

^c^Hard.

^d^Very hard.

^e^Overall systematic bias compared with the ActiGraph device (*P*<.001).

**Table 5:** Accuracy of global positioning system (GPS) location data.

Accuracy radius (m)^a^	Location samples n (%)	Median accuracy radius (m)
≤25	246 (32.3)	19
26-50	307 (40.3)	30
51-75	29 (3.8)	57
76-100	45 (5.9)	96
>100	134 (17.6)	2370
Total	761 (100.0)	30

^a^68% probability of the true position lying within specified radii of the recorded location coordinates.

#### Understanding Geospatial Human Movement

Free-living accelerometry yielded 30,666 records; during this time, Movn logged 761 GPS coordinate pairs. The majority of GPS data (553/761, 72.7%) had acceptable location measurement accuracy (68% probability of true position lying within a 50-m radius of recorded location; [24]) and the median location accuracy radius was 30 m (Table 5); however, almost 20% of GPS location data were characterized by low measurement accuracy (accuracy radius>100 m, median=2370 m).

**Figure 7:**
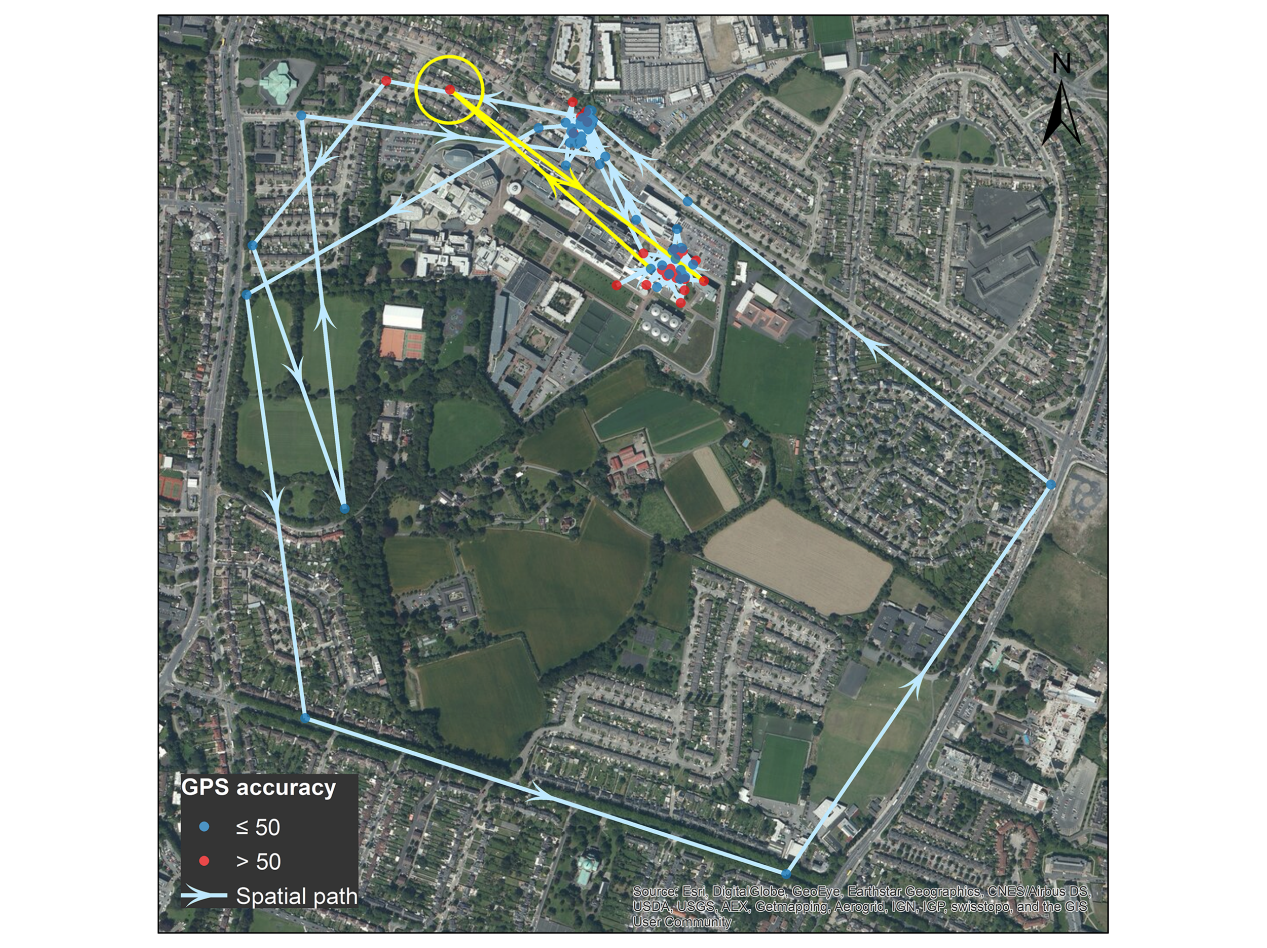
Example of participant location markers and interpolated spatial path.

Figure 7 presents an example of mapped daily location data for 1 participant; blue and red markers indicate accuracy radii of ≤50 m and >50 m, respectively. Straight paths were interpolated between sequential locations; arrows show movement direction. The circled location is likely erroneous given the significant deviation from previous and subsequent locations over a relatively short time and sharp turning angle (see the yellow highlighted path).

**Figure 8:**
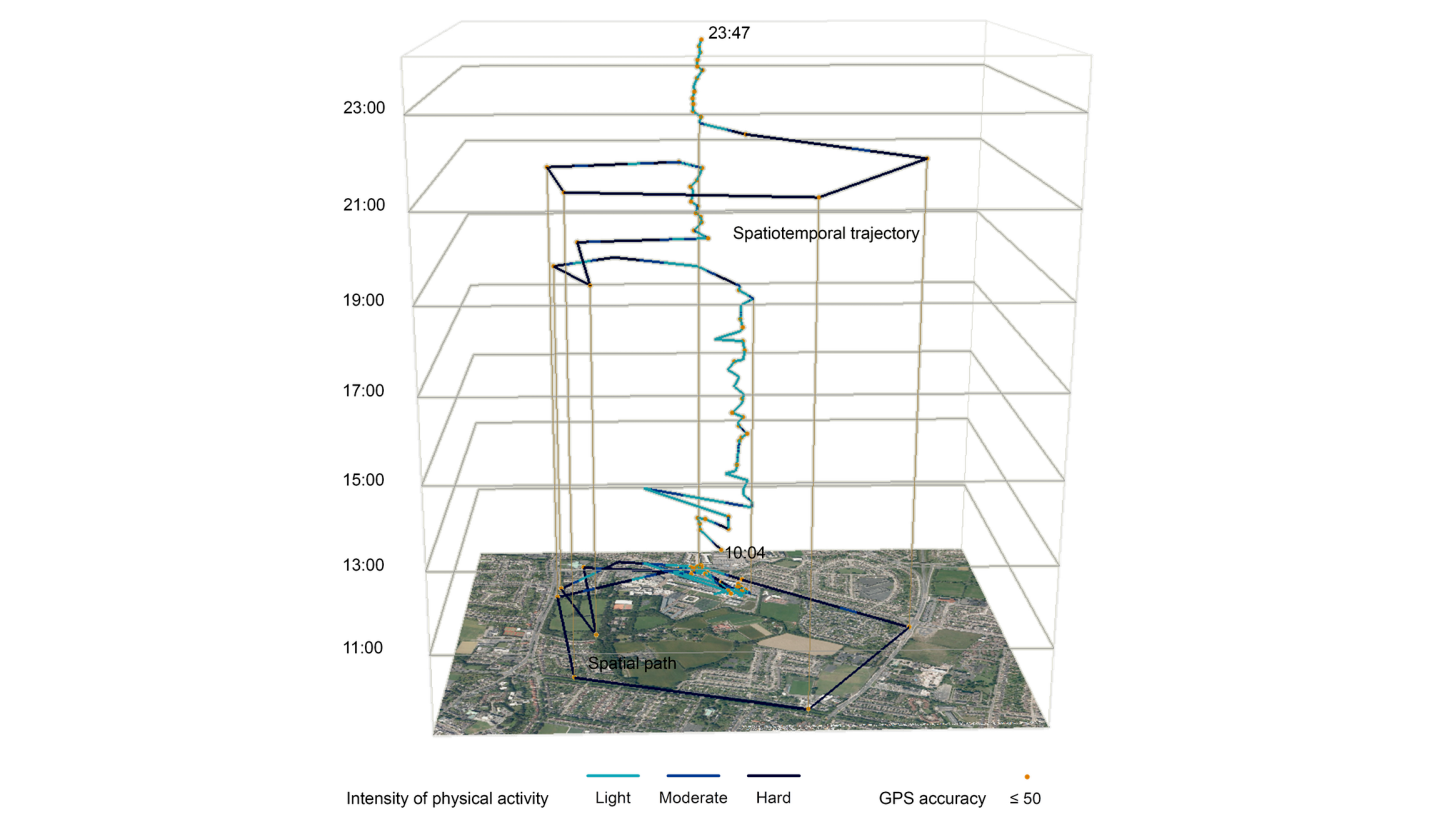
Detailed example of a participant’s daily movement. Position is depicted on the two-dimensional plane, and time is depicted on the vertical plane. Graduated shading of the movement path indicates activity level (dark shading=higher activity level). Orange point markers represent locations with an accuracy of >50 m.

The temporal variability of activity intensity level is not readily displayed by the two dimensions, and dense concentrations of location markers make it difficult to resolve movement direction sequences. To address this, a time-geography approach [25] was used to present an enhanced 3D illustration of activity patterns; position is depicted on a typical 2D plane, time on the vertical plane, and intensity level using graduated shading (Figure 8). Duration, sequence, and movement information can be observed or inferred from such spatiotemporal trajectories; for example, with the exception of the segments that link to less accurate locations (ie, vertices with orange marker points), the main concentration of points and paths indicate that this nominal participant spent the majority of the day (approximately 11:00 AM-17:40 PM) conducting light-intensity activities around a central location. In the evening (approximately 17:40 PM-18:40 PM), the participant traveled a relatively long distance with higher-intensity PA.

## Discussion

### Principal Findings

This study sought to examine the validity of the Movn smartphone app for assessing PA and human movement patterns. In agreement with previous research, Movn activity counts were strongly related to measured EE, and the SEE for the predictive function compared favorably with that reported by Freedson et al [19]. Activity counts associated with thresholds for categorizing intensity levels were lower than those reported by Freedson et al [19], possibly because of differences between algorithms that convert raw accelerometer output signals (ie, voltage) into activity counts. Compared with indirect calorimetry in a controlled laboratory setting, an absence of systematic overall measurement bias, moderate limits of agreement, and very strong correlations demonstrated good agreement for Movn-derived estimates of EE. Agreement was not as strong when using a different model smartphone in the cross-validation model, and this may be explained by differences between integrated solid state accelerometer chips.

Together, these data suggest that Movn could provide acceptable PA measurement precision in controlled laboratory settings; however, greater error variability at higher activity intensity levels suggests that it may be better suited to quantifying general daily PAs than higher intensity exercise training. These findings are comparable with the recent validations of Android smartphone-based activity measurement tools, which demonstrated strong correlations but larger measurement error at higher activity intensity levels when compared with the ActiGraph GTX3 accelerometer [7,8].

During 24 hours of free-living activity, Movn overestimated EE compared with the commonly used and validated ActiGraph accelerometer; biases were, once again, larger and more variable during higher intensity activities. Nonetheless, Movn and reference estimates of EE were strongly related, and these data compare favorably to other data comparing smartphones and the Fitbit Zip wearable sensor with the ActiGraph in free-living settings [6,7]. Differences in measurement units preclude more detailed comparisons of measurement validity.

Interindividual variability revealed in exploratory supplementary analyses indicated the Movn app may be most suitable for group-level monitoring. As smartphone apps can be more feasibly and affordably distributed across large populations than wearable sensors such as the ActiGraph, this approach holds considerable potential for large-scale population-based research.

It was feasible to integrate smartphone-collected geospatial and accelerometry data within a GIS to provide insight into temporal movement patterns (location and intensity). In combination with the potential for large-scale population surveillance, the capability to augment EE data with mapped movement patterns highlights the potential for smartphone-based measurement tools to support novel research evaluating the drivers and effects of interventions and policies that impact PA. However, such large-scale analyses also present challenges for data processing and analytics.

Collectively, this study and previous works highlight the value of smartphones as an acceptable measure of PA in both laboratory-based and free-living contexts. Given their ubiquity, integrated sensor features (accelerometry, gyroscope, inclinometer, and GPS), and passive data collection, smartphones offer additional value compared with existing measurement approaches. Further value is added by the capacity to generate large datasets that could be used to understand temporal, location, and contextual factors that affect PA. Such information could be used to provide point of decision prompts or behavior change strategies to increase PA and decrease sedentary behavior.

The integration of geospatial and accelerometry data within a GIS in this study demonstrates the potential of using smartphones for describing the context (ie, time and location of activity) of human movement patterns. This approach has been demonstrated but has typically relied on separate devices to collect activity and geospatial data, which adds participant burden and limits the duration of observation (7-14 days). Passive smartphone data collection reduces participant burden and permits sustained data collection, for as long as the app is installed and individuals continue to charge their smartphones. This is exemplified by many commercially available smartphone apps that currently harness these features (eg, Moves, Human, and Movn). Smartphones offer pragmatic advantages over stand-alone GPS devices, although the maximum data capture frequency is typically lower (approximately 1 Hz vs 5+ Hz) [26]. Increased sampling frequencies have the potential to improve GPS measurement accuracy but would have a detrimental effect on smartphone battery life and may only offer additional benefits during activities with rapidly fluctuating movement patterns [26]. In this study, the Movn app was configured to record GPS location every 30 min, or when activity was detected via accelerometry to optimize smartphone battery life. More frequent sampling would increase the resolution of geospatial movement patterns but may limit the maximum recording duration because of faster power consumption.

This study has important implications for future research. Smartphones can passively measure PA in large population groups without the use of dedicated measurement tools and provide opportunities to enrich traditional PA measurement with contextualizing temporal and geopositional data. Future work could leverage existing integration between smartphones, smartwatches, and consumer-grade wearable activity trackers to capture PA during periods of smartphone noncarry time. Temporal and positional contextual data provide opportunities to understand peoples’ movement patterns, and this may help to identify and capitalize on optimal opportunities for PA intervention. Utilizing the big data generated by smartphone apps offers opportunities to provide detailed information on how and where people move and whether these patterns are stable over time. If patterns can be predicted, then interventions could be delivered via smartphones to promote PA and reduce sedentary behavior. Geospatial data could be linked with other sensors or data sources via the Internet of Things to provide “just-in-time” interventions such as promoting active transport options (nearby cycling or walking routes) rather than driving a motor vehicle. Such an approach would be consistent with the notion of smart city research, which harnesses the Internet of Things for public and environmental health surveillance [9].

Smart cities focus investment on digital infrastructure, including information and communication technology rather than traditional physical infrastructure. Whereas smart city technologies typically focus on system efficiencies (traffic and waste management, etc), they offer potential to promote PA and health [9,27]. To fully maximize the benefits of smartphone data, it will be necessary to develop big data analytical methods to extract, process, and interpret large quantities of data. To achieve this, PA researchers need to develop expertise in these techniques or collaborate with people who have appropriate expertise in big data processing and analytics.

### Strengths and Limitations

Strengths of this study include the use of indirect calorimetry as a criterion reference for the validation of EE estimation, the use of a cross-validation sample to assess the validity of EE estimation on different smartphones, and the integration of geospatial and accelerometry data within a GIS. A limitation of this research relates to the data reduction methods of smartphone data. Unlike research-grade accelerometers, there are no established methods for managing smartphone accelerometer data. Periods of negative EE measurement bias may have indicated smartphone noncarry time; however, as methods for classifying phone and individual inactivity have yet to be determined, it was not possible to validate this assumption. We applied criteria typically used for accelerometers (removing >60 min of consecutive zero values), which may have assisted with agreement between methods but do not differentiate smartphone noncarry time from true inactivity. Further research is needed to characterize data patterns that can distinguish these use patterns, and this will have a significant impact on how smartphone data are interpreted. The increasingly common inclusion of embedded gyroscopic sensors could help to overcome this problem. It should be noted that stand-alone wearable accelerometers are also subject to this limitation, and prolonged nonwear time may result in greater data loss than intermittent smartphone noncarry time. Researchers should consider these limitations and select the type of measurement tool that best suits their experimental objectives. Additional limitations include the number and characteristics of participants and the limited period of free-living activity monitoring. As the primary sampling unit in this study is sensor observations not individuals (ie, 50 Hz accelerometer data), the study is appropriately powered to achieve validation objectives. Because participants were healthy adults, these results may not generalize to populations with musculoskeletal limitations or medical conditions that modify the energetic demands of comparable PAs. Finally, the short 24-hour free-living validation period may limit the range of activities and activity intensity levels included in the free-living analysis. Although high frequency sensor sampling enables ample statistical power for validation objectives, future research may consider longer data collection periods that may include a wider range of activities.

### Conclusions

The Movn smartphone app provided valid measurement of physical activity EE at low and moderate activity intensity levels; however, measurement validity was reduced during higher intensity activities. Given their ubiquity, integrated sensors, passive data collection, and potential to connect with external data streams, smartphones provide an ideal opportunity to enhance understanding of the nature and context of human movement, particularly at a population level. This presents future challenges for data processing and analytics, as well as opportunities to inform novel, responsive, and individualized interventional strategies.

## Abbreviations

CO_2_: carbon dioxide
EE: energy expenditure
GIS: geographic information software
GPS: global positioning system
ICC: intraclass correlation coefficient
MET: metabolic equivalent of task
O_2_: oxygen
PA: physical activity
SPSS: Statistical Package for the Social Sciences
3D: three-dimensional
2D: two-dimensional
V̇O_2_: oxygen uptake
WHO: World Health Organization
